# Effect of illness perceptions on asthma control and quality of life amongst adult outpatients with asthma in China

**DOI:** 10.1186/s40359-023-01097-3

**Published:** 2023-03-12

**Authors:** Qingqing Cai, Meiling Jin, Xiaoyu Li, Jieqing Zhang, Qing Xu, Ling Ye, Qianzhou Lyu

**Affiliations:** 1 Department of Pharmacy, Zhongshan Hospital, Fudan University, Shanghai, 200032 People’s Republic of China; 2 Department of Respiratory Medicine, Zhongshan Hospital, Fudan University, Shanghai, 200032 People’s Republic of China; 3 Department of Allergy, Zhongshan Hospital, Fudan University, Shanghai, 200032 People’s Republic of China

**Keywords:** Asthma, Illness perception, Medication adherence, Quality of life, Asthma control, Common-sense model

## Abstract

**Objective:**

To investigated the influence of illness perceptions and other risk factors related to poor asthma control and quality of life in adult outpatients with asthma in China.

**Methods:**

Patients with a confirmed asthma diagnosis were recruited from the outpatient clinic at Zhongshan Hospital, Fudan University in Shanghai. Sociodemographic, psychological, and asthma related variables were assessed in all participants. Patients’ illness perceptions, medication adherence, asthma control, and quality of life were assessed using validated questionnaires, such as the Brief Illness Perception Questionnaire, Medication Adherence Rating Scale (MARS-A), the Asthma Control Test, and the Mini Asthma Quality of Life Questionnaire. Multiple linear regressions and logistic regressions were used to examine the associations between illness perceptions, medication adherence behaviors, and disease outcome (i.e., asthma control and quality of life).

**Results:**

A total of two hundred thirty-one (231) outpatients with asthma were included in this cross-sectional study, 80 of whom (34.6%) had asthma that was uncontrolled. Patients who perceived their life (β = − 0.197,* p* < 0.001) and emotions (β = − 0.294,* p* < 0.001) as severely affected by the illness were more likely to have a lower quality of life, findings that were statistically significant. Also, patients who believed they had a higher degree of personal control over their illness (β = 0.333,* p* < 0.001), and had better medication adherence (β = 0.250,* p* < 0.001) were found to have a better quality of life.

**Conclusion:**

Our study indicated that illness perceptions and medication adherence have a significant impact on disease outcome. Both of these factors should be considered when determining the best health care practices or constructing a predictive intervention model for patients with uncontrolled asthma.

## Introduction

Asthma is a chronic condition affecting an estimated 45.7 million adults in China [1]. The disease is characterized by chronic airway inflammation, which causes various respiratory symptoms that can have a severe impact on a patient’s daily life [2, 3]. Sub-optimal asthma control is also associated with increased cost of hospitalization and decreased quality of life [4]. Despite the availability of effective asthma controller medications in China, only 28.7% and 45.0% of patients in the country have achieved complete or partial asthma control, respectively, based on the Global Initiative on Asthma (GINA) criteria [5].

Adequate self-management is crucial for achieving and maintaining optimal asthma control and health outcomes [6, 7]. To achieve good self-management, a patient’s knowledge of the disease and coping strategies are the two key determinants for positive health outcomes [8]. The critical role of illness perceptions about asthma in determining health outcomes has been based on the Leventhal’s common-sense model [9, 10], which describes how patients react to their illness through both cognitive and emotional representations. These representations form patients’ motivation to take specific strategies to manage their emotions and improve asthma control [10, 11].

Previous research has suggested a tight link between quality of life and illness perceptions [12–14]. Specifically, patients with more positive illness perceptions are expected to report a better quality of life, whereas negative ones are associated with lower quality of life [13]. Recent research also indicates that positive illness perceptions are associated with better adherence, which in turn, results in better disease control and quality of life [15]. Conversely, patients’ non-adherence to medication contributes to poor clinical outcome [16, 17]. In particular, poor adherence to asthma controller therapy may contribute to progressive decline in lung function [18], increased risk of severe asthma exacerbations [19], and decreased quality of life [20].

Our previous study showed that 42.3% of participants were non-adherent to their inhaled corticosteroid (ICS) medication [21]. In addition, this work suggested that illness perception has a strong influence on self-reported medication adherence in adult patients with asthma in China, but little is known about its impact on asthma control and quality of life on those patients. Improvements in clinical outcome and quality of life in Chinese patients with asthma are important, as they may bring great benefit to the patients, as well as their families. With this background, the aim of the present study is to investigate the influence of illness perceptions and other risk factors related to poor asthma control and quality of life in Chinese adult outpatients with asthma.

## Materials and methods

### Study design

A cross-sectional study was performed between October 2018 and September 2019 in the asthma outpatient clinic of Zhongshan Hospital, Fudan University in Shanghai. Ethical approval was granted by the the Ethics Committee of Zhongshan Hospital, Fudan University. The inclusion criteria were: (1) Adult patients (aged ≥ 18 years) with a confirmed physician-diagnosed asthma; (2) Patients treated with an ICS alone or ICS plus long-acting beta 2-agonist (LABA) combinations for at least one month. The exclusion criteria were patients with severe pulmonary disease or chronic obstructive pulmonary disease, and those who are unable to read and communicate. Eligible patients signed informed consent forms before enrollment.

To achieve higher response rates, questionnaire completion rates, and reduce cognitive burden for participants [22], data were collected by questionnaires via a face-to-face interview performed by a trained pharmacist. FEV1% (forced expiratory volume in 1 s) predicted and FENO (fractional exhaled nitric oxide measurement) data in the year prior to inclusion were acquired by retrospectively reviewing medical records in our institution.

### Measures

#### Brief illness perception questionnaire (B-IPQ)

Patients’ illness perceptions were measured with the Chinese validated version of B-IPQ, which is a nine-item questionnaire assessing a patient’s cognitive and emotional representations of illness [23]. Items include perception of illness identity (How much do you experience symptoms from your illness?), consequences (How much does your illness affect your life?), timeline (How long do you think your illness will continue?), comprehension (How well do you feel you understand your illness?), concerns (How concerned are you about your illness?), personal control (How much control do you feel you have over your illness?), treatment control (How much do you think your medication can help your illness?), and emotional response (How much does your illness affect you emotionally?) [24]. The ninth item is an open-ended question used to assess the perceived cause of illness. Each item is checked on an 11- point Likert scale from 0 to 10, with higher scores indicating stronger endorsement of that dimension. The Chinese B-IPQ exhibited good test–retest reliability (Pearson correlation coefficient of 0.42 to 0.75).

#### Medication adherence report scale for asthma (MARS-A)

Medication adherence to daily ICS treatment was measured using Chinese version of MARS-A. The MARS-A is a 10-item scale measuring both intentional and unintentional medication non-adherence [25]. Scores of each item are rated on a five-point Likert scale (1 “always” to 5 “never”). The MARS-A score was calculated as the mean score of all items, with an average score ≥ 4.5 indicating high levels of adherence [26]. This version of MARS-A displayed excellent internal consistency with a Cronbach’s alpha (α) of 0.93.

#### Asthma control test (ACT)

The ACT is a self-reported measure for the assessment of a patient’s level of asthma control in the preceding four weeks [27]. This 5-item questionnaire consists of the following subscales: the impact of asthma on daily functioning; asthma symptoms; rescue medications use; and patient’s subjective self-rating of their asthma control. Each question is scored from 1 to 5, and the sum of responses is calculated. ACT scores ≥ 20 identifies patients as having “well controlled” asthma, while scores ≤ 19 identifies patients with “uncontrolled” asthma [27].

#### The mini asthma quality of life questionnaire (mini-AQLQ)

The Chinese validated version of mini-AQLQ was used to measure quality of life in patients with asthma during the preceding two weeks [28]. It is a 15-item questionnaire covering four domains: activity limitation, emotional function, environmental stimuli and symptoms. All items are scored on a seven-point Likert scale, ranging from 1(worst) to 7 (best), with a lower score indicating a more impaired quality of life. The overall mini-AQLQ score and score of each domain were calculated by averaging items on each subscale.

#### Statistical analysis

Data analysis in the current study was performed using IBM SPSS 23.0 program. Continuous variables were summarized using mean ± standard deviation (SD), and categorical variables were presented as frequencies and percentages. Groups were compared using two-sided t-test, chi-square test, or Wilcoxon rank sum test, as appropriate. The correlation between illness perception and asthma control and quality of life were measured using Spearman correlation.

Univariate logistic regression analysis was applied to evaluate associations between asthma control and independent parameters [age, gender, duration of ICS use, duration of asthma (> 10 years), FEV1% predicted, illness perception items and medication adherence]. Multivariate logistic regression analysis was conducted by manual input variables, with the final model including variables that had *p* values < 0.10 in the univariate analysis. Odds ratios (OR) and 95% confidence interval (CI) were presented for all models. Linear regression was used to investigated factors associated with factors associated with quality of life. Predictor variables included patients’ age, gender, duration of ICS use, value of the illness perception questionnaire, and medication adherence. Results were considered significant when the *p* value was < 0.05.

## Results

### Sample characteristics

Demographic and clinical characteristics of the participants are presented in Table 1. A total of 231 adult outpatients with asthma were included in the survey, 80 of whom (34.6%) had uncontrolled asthma. The mean ACT scores were 23.11 ± 1.54 and 15.45 ± 3.20 for the well-controlled group and uncontrolled group, respectively (*p* < 0.001). The mean age was 43.20 ± 14.68 years in the well-controlled group versus 48.50 ± 14.22 in the uncontrolled group. Patients in the uncontrolled group were more likely to have a longer duration of asthma (*p* = 0.002), longer regular ICS treatment (*p* = 0.039), and lower medication adherence (*p* = 0.006).

**Table 1:** Baseline of study subjects

Variables	Well controlled group (n = 151)	Uncontrolled group (n = 80)	*p*-value
Age, years, mean ± SD	43.20 ± 14.68	48.50 ± 14.22	0.009
Gender (N, %)			0.483
Male	65 (43.0)	30 (37.5)	
Female	86 (57.0)	50 (62.5)	
Duration of regular ICS treatment, years, mean ± SD	3.66 ± 4.42	5.14 ± 5.22	0.039
Age of asthma onset (N, %)			0.524
< 18-year old	39 (25.8)	18 (22.5)	
18–45 years of age	82 (54.3)	41(51.2)	
> 45-year old	30 (19.9)	21(26.3)	
Duration of asthma (N, %)			0.002
< 5 years	86 (57.0)	27 (33.8)	
5–10 years	24 (15.8)	14 (17.5)	
> 10 years	41 (27.2)^a^	39 (48.7)^a^	
Education level (N, %)			0.072
Primary education	6 (4.0)	5 (6.3)	
Secondary education	14 (9.3)	16 (20.0)	
Senior high education	20 (13.2)	12 (15.0)	
University education	111 (73.5)	47 (58.8)	
Occupation (N, %)			0.043
Physical labor	7 (4.6)	6 (7.5)	
Office work	84 (55.6)	39 (48.8)	
Participants unemployed	50 (33.1)	35 (43.8)	
Student	10 (6.6)	0 (0)	
Current maintenance treatment			0.752
ICS	8 (5.3)	3 (3.8)	
ICS + LABA	143 (94.7)	77 (96.3)	
MARS-A score, mean ± SD	4.32 ± 0.91	3.83 ± 1.22	0.006
FEV1% predicted, mean ± SD	84.13 ± 23.82	72.89 ± 25.10	0.014
FENO ppb, mean ± SD	48.28 ± 37.46	49.19 ± 28.17	0.357
ACT score	23.11 ± 1.54	15.45 ± 3.20	0.000

ACT scores ≥ 20 indicated “well controlled” asthma, while scores ≤ 19 indicated “uncontrolled” asthma

FEV1% predicted and FENO data were acquired by retrospectively reviewing medical records in our institution in the year prior to inclusion

*SD* Standard deviation; *ICS* Inhaled corticosteroid; *LABA* Long-acting beta 2-agonist; *FEV1* Forced expiratory volume in 1 s; *FENO* Fractional exhaled nitric oxide measurement; *ACT* Asthma control test

^a^ *p* < 0.05

### Correlation between illness perception components, medication adherence, asthma control, and quality of life

As shown in Table 2, most illness perception items were correlated with ACT scores and AQLQ-overall scores. ACT scores were negatively correlated with consequences, identity, and emotional representation (r = − 0.372, − 0.352 and − 0.282, respectively, *p* < 0.01), while personal control and treatment control over illness were positively correlated (r = 0.306 and 0.185, respectively, *p* < 0.01). There were negative correlations between consequences, identity, illness concern, emotional representation, and AQLQ-overall scores (r = − 0.479, − 0.316, − 0.239 and − 0.437, respectively, *p* < 0.01), but there was a significant positive correlation with personal control (r = 0.352,* p* < 0.01).

Significant positive correlations were also found between medication adherence and ACT scores (r = 0.271,* p* < 0.01); medication adherence and AQLQ-symptoms score (r = 0.254,* p* < 0.01); medication adherence and AQLQ-emotion score (r = 0.193,* p* < 0.01); and medication adherence and AQLQ-overall scores (r = 0.234,* p* < 0.01). Moreover, asthma control was strongly correlated with quality of life (r = 0.650, *p* < 0.001).

**Table 2:** Correlations between illness perception components, asthma control and quality of life

	ACT	AQLQ-activity	AQLQ-symptoms	AQLQ-emotion	AQLQ-environment	AQLQ-overall
BIP-1 Consequences-How much does your illness affect your life?	− 0.372**	− 0.375**	− 0.371**	− 0.463**	− 0.166*	− 0.479**
BIP-2 Timeline-How long do you think your illness will continue?	0.026	0.087	0.053	0.023	− 0.103	− 0.011
BIP-3 Personal Control-How much control do you feel you have over your illness?	0.306**	0.397**	0.305**	0.232**	0.076	0.352**
BIP-4 Treatment control -How much do you think your treatment can help your illness?	0.185**	0.178**	0.177**	− 0.012	− 0.041	0.078
BIP-5 Identity-How much do you experience symptoms from your illness?	− 0.352**	− 0.355**	− 0.244**	− 0.264**	− 0.073	− 0.316**
BIP-6 Concern-How concerned are you about your illness?	− 0.080	− 0.116	− 0.073	− 0.271**	− 0.162*	− 0.239**
BIP-7 Comprehension-How well do you feel you understand your illness?	0.075	0.099	0.128	0.036	0.021	0.083
BIP-8 Emotional Response-How much does your illness affect you emotionally? (e.g., does it make you angry, scared, upset or depressed	− 0.282**	− 0.165*	− 0.323**	− 0.559**	− 0.212**	− 0.437**
ACT scores		0.502**	0.783**	0.427**	0.244**	0.650**
Medication adherence	0.271**	0.121	0.254**	0.193**	0.097	0.234**

*ACT* Asthma control test; *BIP* Brief illness perception; *AQLQ* Asthma quality of life questionnaire

* p < 0.05 ** p < 0.01

### Factors associated with uncontrolled asthma

Table 3 shows the results of logistic regression analysis. The multiple regression analysis shows that higher personal control was associated with lower odds (OR 0.75, 95% CI 0.62–0.90,* p* = 0.003). Similarly, higher medication adherence was associated with lower odds (OR 0.59, 95% CI 0.40–0.88, *p* = 0.009). Longer duration of asthma was associated with higher odds (OR 3.07, 95% CI 1.04–9.05, *p* = 0.042). Patients with poorer medication adherence, longer disease duration, and those who believe they have lower personal control over their illness were more likely to have asthma that was uncontrolled.

**Table 3:** Factors associated with uncontrolled asthma in adults in China

	Univariate logistic regression		Multivariate logistic regression		
	Odds ratio (95% confidence interval)	*P*-value	Odds ratio (95% confidence interval)		*P*-value
Age	1.02 (1.01–1.04)	0.010	1.01 (0.98–1.04)		0.698
Gender	0.79 (0.46–1.38)	0.415			
Duration of ICS use	1.06 (1.01–1.13)	0.031	0.95 (0.85–1.05)		0.287
Duration of asthma					
> 10 years	3.03 (1.64–5.61)	0.000	3.07 (1.04–9.05)		0.042
FEV1% predicted	0.98 (0.97–0.99)	0.009	1.00 (0.98–1.02)		0.831
BIP-1 Consequences	1.38 (1.22–1.56)	0.000	1.17 (0.95–1.45)		0.135
BIP-2 Timeline	1.04 (0.95–1.13)	0.449			
BIP-3 Personal control	0.77 (0.67–0.87)	0.000	0.75 (0.62–0.90)		0.003
BIP-4 Treatment control	0.91 (0.80–1.03)	0.128			
BIP-5 Identity	1.36 (1.21–1.53)	0.000	1.05 (0.86–1.29)		0.620
BIP-6 Concerns	1.04 (0.91–1.20)	0.533			
BIP-7 Comprehension	0.98 (0.88–1.08)	0.670			
BIP-8 Emotional response	1.17 (1.06–1.29)	0.002	1.15 (0.97–1.36)		0.105
Medication adherence	0.65 (0.50–0.84)	0.001	0.59 (0.40–0.88)		0.009

*ACT* Asthma control test; *BIP* Brief illness perception; *ICS* Inhaled corticosteroid; *FEV1* Forced expiratory volume in 1 s

### Factors associated with quality of life

Table 4 shows that patients’ illness perceptions, such as consequences, personal control, and emotional response were significantly linked to patients’ quality of life in the multiple linear regression. The more severe that patients perceived their illness to be, the more it affected their life (β = − 0.197,* p* < 0.001). Also, the higher they scored in emotional response to illness (β = − 0.294,* p* < 0.001), the more likely they were to have lower quality of life. Patients who believed they had a higher degree of personal control over their illness (β = 0.333,* p* < 0.001) and higher medication adherence (β = 0.250,* p* < 0.001) were found to have a better quality of life.

**Table 4:** Linear regression for factors associated with quality of life (AQLQ-overall total score)

	Univariate regression			Multiple regression		
	Coefficient	95% CI	*p*-value	Coefficient	95% CI	*p*-value
Age	− 0.142	− 0.018, − 0.001	0.038			
Duration of ICS use	− 0.123	− 0.052, 0.002	0.073			
FEV1% predicted	0.242	0.003, 0.016	0.003			
BIP-1 Consequences	− 0.485	− 0.208, − 0.126	0.000	− 0.197	− 0.120, − 0.018	0.008
BIP-2 Timeline	0.027	− 0.033, 0.049	0.695			
BIP-3 Personal control	0.358	0.098, 0.204	0.000	0.333	0.083, 0.187	0.000
BIP-4 Treatment control	0.100	− 0.015, 0.103	0.145			
BIP-5 Identity	− 0.316	− 0.158, − 0.067	0.000			
BIP-6 Concerns	− 0.198	− 0.150, − 0.030	0.004			
BIP-7 Comprehension	0.092	− 0.015, 0.081	0.181			
BIP-8 Emotional response	− 0.425	− 0.170, − 0.094	0.000	− 0.294	− 0.139, − 0.051	0.000
Medication adherence	0.273	0.128, 0.360	0.000	0.250	0.107, 0.327	0.000

*AQLQ* Asthma quality of life questionnaire; *BIP* Brief illness perception; *ICS* Inhaled corticosteroid; *FEV1* Forced expiratory volume in 1 s; *CI* Confidence interval

## Discussion

This study set out to evaluate the effects of illness perceptions and other factors on asthma control and quality of life amongst adult outpatients with asthma in China. The key finding was that both patients’ illness representations and medication adherence were associated with asthma control and quality of life.

In this study, 34.6% patients had uncontrolled asthma. This was consistent with the finding in a study by Chiu et al., in which 64% of Asian patients had well-controlled asthma [29]. Our study also showed that patients with uncontrolled asthma were more likely to be older, have longer duration of the disease, and longer ICS treatment. Similarly, a previous study reported that older age and longer duration of asthma were the factors significantly influencing sub-optimal control of asthma [30]. In addition, medication adherence to ICS treatment was found to be associated with disease control, such as asthma control and quality of life in our study, a result consistent with previous studies [15–17, 31], which showed that patients with low adherence had lower baseline ACT scores [31].

Most illness perceptions were significantly correlated with asthma control. Personal control remained significant in multiple regression analyses after adjusting for the covariates that were significant in the univariate analysis. This result was in line with previous studies, which showed that greater perceived personal control over asthma was significantly associated with better disease control [32, 13]. It is postulated that individuals who have a higher degree of personal control might perceive less stress, which in turn, is associated with a positive impact on asthma control [33, 34]. In addition, Achstetter et al. showed that illness perception reflecting personal control predicts the change in asthma control achieved during a short-term intervention [34].

A similar pattern was seen with quality of life, in which patients with positive illness perceptions tended to report a better quality of life. Higher consequences and emotional representation were strongly associated with lower quality of life, whereas higher personal control was related to higher quality of life. Kosse et al. showed that adolescents who reported more severe consequences from asthma and perceived themselves to have less personal control over asthma, while also scoring higher in emotional reaction to the disease were more likely to report a decreased quality of life [15].

The results of the present study have clinical implications. It is suggested that improving patients’ medication adherence behavior and illness perceptions might improve the health-related outcomes in adults with asthma. Since a more positive illness perception, less emotional representation, and higher medication adherence are related to better disease control and quality of life in patients with asthma, we postulate that interventions for those patients who are in low medication adherence and experience negative illness perceptions may benefit from targeting these factors. This ‘paradigm’ indeed has proved to be valid. Petrie et al. showed that targeted text message intervention based on an assessment of patients’ illness and medication belief can improve adherence with preventer inhaler medication [35]. An interactive mobile health (mHealth) intervention based on the Common Sense Model of Self-regulation significantly increased medication adherence in adolescents with asthma having poor adherence rates at baseline [36]. Given the empirical evidence that changing patients’ illness perceptions might have a positive effect on patient’s adherence and disease outcome, further studies addressing patients’ illness perception are needed in order to achieve optimal asthma control and quality of life.

### Limitations

The present study has some limitations. First, the cross-sectional design of the study precluded us from evaluating the causal relationship between illness perceptions, treatment adherence, disease control and quality of life. However, this study paves the way for future interventional research in China targeting the value in changing patients’ illness perceptions and medication adherence to improve asthma control and quality of life in patients with asthma. Second, participants were recruited from specialist outpatient clinics rather than respiratory outpatient clinics using convenience sampling, which might induce a sample selection bias and an overestimated adherence rate in our study population. Third, the questionnaires were designed to be self-administered, however, data collection in our study was carried out by face-to-face interviews. Compared to self-administered mode, face-to-face interview mode could result in higher social desirability bias [22]. Interview respondents are more likely to give high or positive rating of their health than self-administered respondents. But most researches comparing self-administered and face-to-face interview mode has reported little or no systematic difference in health-related indicators [37]. Forth, medication adherence was based on self-reported data, which may be vulnerable to recall bias. Future studies could use either pharmacy claims data or electronic medication prescription monitors as a measure of adherence to objectively assess adherence. Fifth, it should be noted that the research approach used a Western biopsychosocial model, the concept of illness of perception is relatively new to most Chinese patients, however, emotion and health are a combination of universal biology and specific culture.

## Conclusion

Our study found that most illness perceptions were significantly correlated with asthma control and quality of life. Patients with more positive illness perceptions, less emotional representation, and higher medication adherence tended to report a better quality of life and asthma control. These findings suggest that self-management interventions targeting the illness perceptions and medication adherence of patients might lead to improved asthma control and better quality of life.

## Data Availability

The datasets used during the current study available from the corresponding author on reasonable request.
